# Supplementary material for: N6-Methyladenine Progressively Accumulates in Mitochondrial DNA during Aging
Source: Int J Mol Sci. 2023 Oct 3;24(19):14858. doi: 10.3390/ijms241914858 (PMC10573865; doi:10.3390/ijms241914858)
Supplement: Supplementary file 1 [file ijms-24-14858-s001.zip › ijms-2570000-supplementary.pdf]

Supplementary materials

Extended Figure S1.

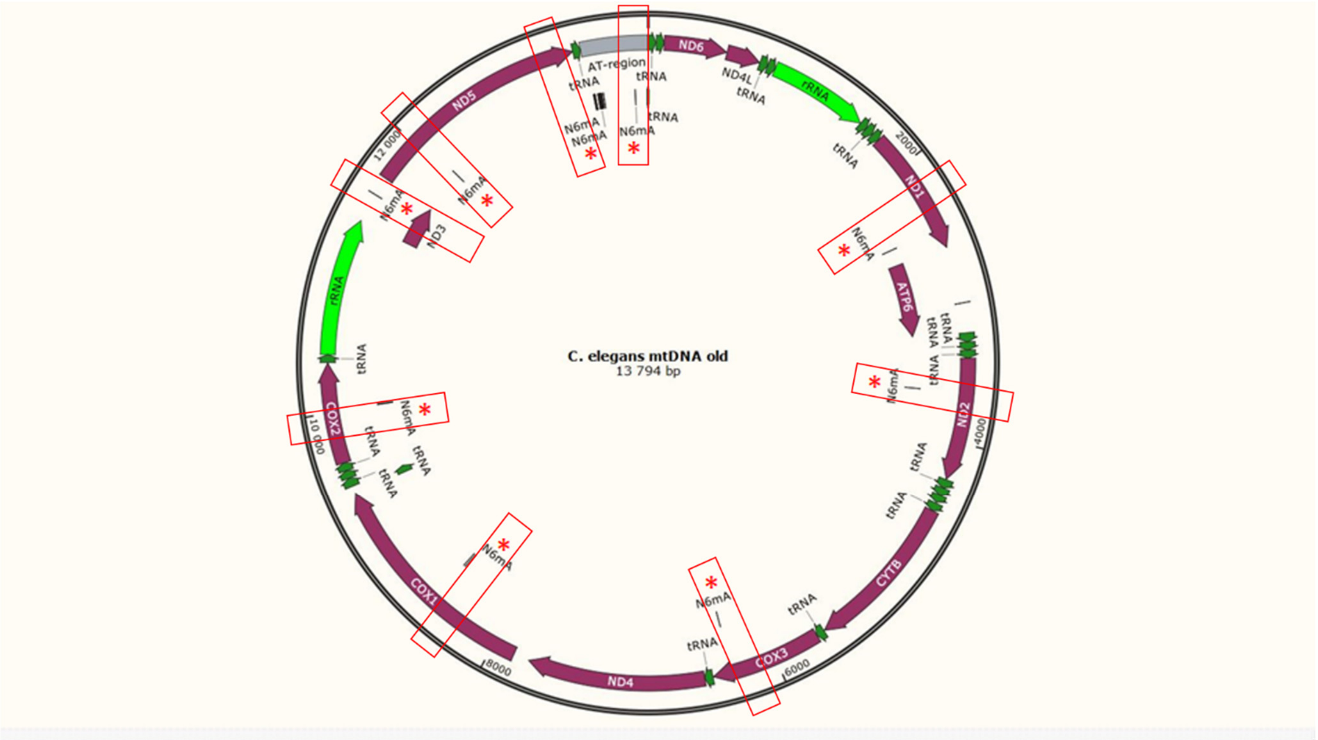

Extended Figure S1. SMRT sequencing identified 6mA sites in the *C. elegans* mitochondrial genome isolated at the adult stage of day 5. 6mA sites are indicated by red asterisks. These sites scatter almost evenly along the mtDNA.

Extended Figure S2.

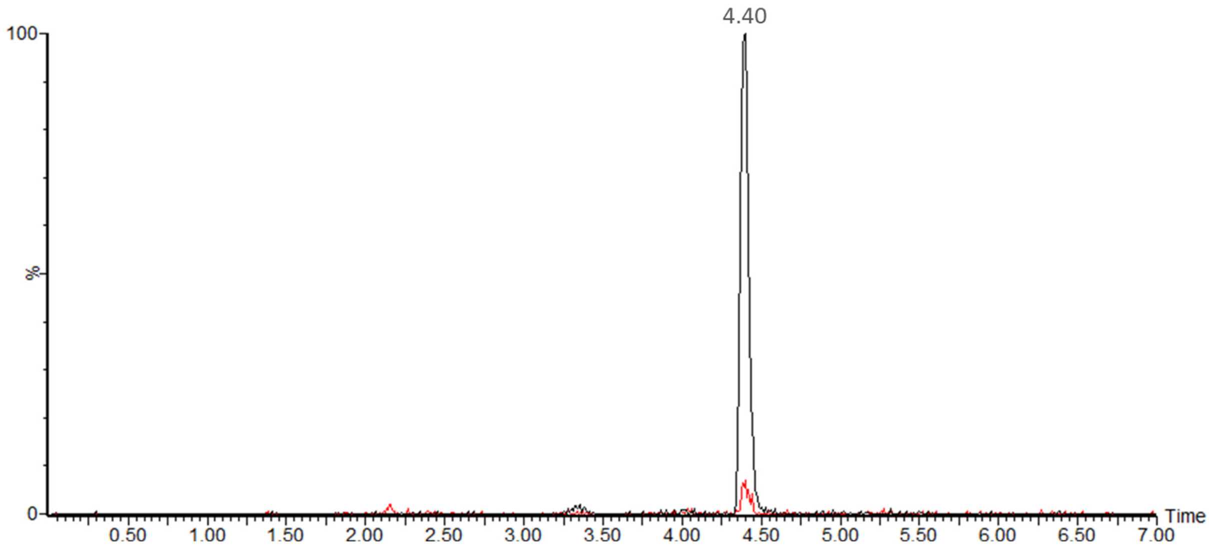

Extended Data Figure S2. LC-MS/MS chromatogram of 6mA levels in the mtDNA isolated from nematodes at adult stages of days 1 and 7. Figure shows the selected ion chromatograms of *m/z* 150.08 from the MS/MS fragmentation of *m/z* 266.1 at a transfer collision energy of 17V. mtDNA was isolated from genomic samples. The young sample at adult age of day 1 (red line) shows basal ("noise") levels of 6mA (0.55 fmol/1000 ng DNA). The aged sample at adult age of day 7 (black line) displays much higher 6mA levels (3.81 fmol/1000 ng DNA). Optimized settings of the Waters Select Series IMS mass spectrometer used for the LC-MS/MS detection of 6mA were as follows:
Capillary voltage: 2.80 kV
Cone voltage: 20 V
Source offset: 30 V
Source temperature: 120 ºC
Cone gas flow: 20 L/hour
Desolvation gas flow: 800 L/hour
Desolvation temperature: 400 ºC
Nebuliser gas flow: 6 bar
Body gradient: 10 V
Head gradient: 20 V
Ion guide 1 offset: 3.0 V
Ion guide 2 offset: 0.3 V
Quadrupole ion energy: 0.4 V
Pre-filter: 2.0 V
Trap CE: 6.0 V
Transfer CE: 17.0 V
Trap TW velocity: 300 m/s
Trap TW pulse height: 1.0 V
Trap Entrance: 2.0 V

Trap bias: 2.0 V
Post Trap gradient: 1.0 V
Post Trap bias: 35.0 V
Collision gas: 3.5 ml/min
StepWave RF: 200 V
Ion guide RF: 300 V
Transfer RF: 200 V
Start mass: m/z 100
End mass: m/z 280
Scan time: 0.5 s
Optic mode: V-Mode
Polarity: Positive

Extended Data Figure S3.

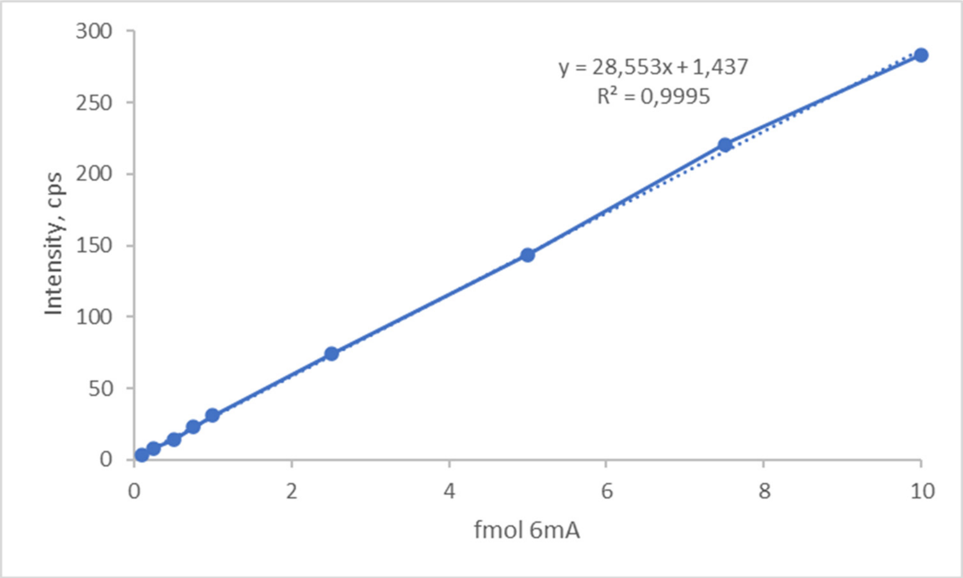

Extended Data Figure S3. Calibration of the LC-MS/MS analysis. Linear calibration between 0.1 amol and 10 fmol was applied using synthetic 6mA. Intensity measured in counts per second (cps). For calibration we used the linear regression method. The dotted line indicates the regression line, where a good correlation (R^2>0,999) was observed. R^2 is the square of the correlation, it measures the proportion of variation in the dependent variable that can be attributed to the independent variable.

Extended Figure S4.

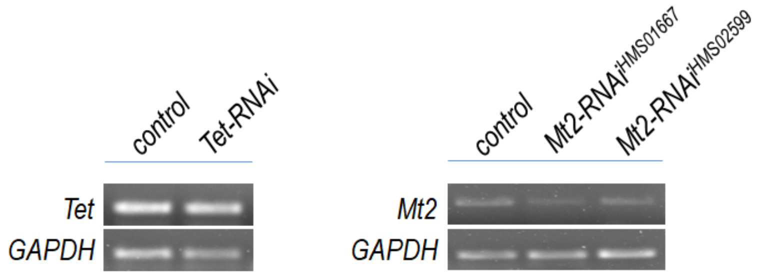

Extended Data Figure S4. Transcript levels in *Tet-RNAi* and *Mt2-RNAi* flies. *Drosophila* RNAi strains containing UAS responder lines were crossed with *Tub-Gal4* driver lines. *GAPDH* was used as an internal control. Control strain refers to *w1118* genotype. *Tet*-RNAi did not decrease *Tet* transcript levels. The RNAi construct is miRNA-based, so it can cause a translational block. Animals were maintained at 25°C. Both *Mt2*-RNAi constructs significantly decreased *Mt2* transcript levels. Animals were kept at 29°C.

Extended Data Table S1. Statistical data.

Background	Primer	Adult age (days)	Trials	Mean of relative 6mA level	±SD	One -way ANOVA with Tukey Post Hoc Test, P value	Significance level
**Figure 2**							
C. elegans wild-type	*Ce_mito3*	1	3	1.000	0.344	control	
		4	3	2.589	0.490	vs. 1. day P=0.0041	**
		7	3	4.593	0.178	vs. 4. day P=0.0057	**
		9	3	5.820	0.092	vs. 7. day P=0.1238	NS
		12	3	7.636	0.136	vs. 9. day P=0.0137	*
	Ce_mito4	1	3	1.000	0.119	control	-
		4	3	3.033	0.259	vs. 1. day P<0.001	***
		7	3	4.955	0.152	vs. 4. day P<0.001	***
		9	3	6.448	0.201	vs. 7. day P<0.001	***
		12	3	7.902	0.232	vs. 9. day P<0.001	***
**Figure 2**							
C. elegans daf-2	*Ce_mito3*	1	3	1.000	0.149	control	-
		4	3	2.525	0.938	vs. 1. day P=0.2158	NS
		9	3	3.655	1.043	vs. 4. day P=0.4934	NS
		12	3	5.101	0.770	vs. 9. day P=0.2589	NS
		16	3	6.920	0.712	vs. 12. day P=0.1040	NS
		20	3	8.258	0.644	vs. 16. day P=0.1040	NS
	Ce_mito4	1	3	1.000	0.566	control	
		4	3	2.414	0.402	vs. 1. day P=0.0026	**
		9	3	4.340	0.326	vs. 4. day P=0.0008	***
		12	3	5.254	0.289	vs. 9. day P=0.0038	**
		16	3	7.193	0.583	vs. 16. day P=0.0205	*
**Figure 4A'**							
D. melanogaster	*Dm_mito2*	1	5	1.000	0.086	-	-
		10	5	4.351	0.621	vs. 1. day P=0.0019	**

		20	5	7.001	1.175	vs. 10. day P=0.0156	*
		30	5	25.394	1.939	vs. 20. day P<0.0001	***
		40	5	8.470	1.211	vs. 30. day P<0.0001	***
	Dm_mito4	1	2	1.000	0.064	-	-
		10	2	2.041	1.174	-	-
		20	2	3.621	0.860	-	-
		30	2	10.029	0.069	-	-
		40	2	4.962	0.135	-	-

Figure 5B

**Background**	**Primer**	**Adult age (years)**	**Trials**	**Mean of relative 6mA level**	**±SD**	**One -way ANOVA with Tukey Post Hoc Test, P value**	**Significance level**
dog	*Cl_mito2*	1	3	1.000	0.245	-	-
		4	6	2.521	0.278	vs. 1. year P<0.001	***
		7	3	3.315	0.365	vs. 4. year P=0.007	**
		12	3	5.358	0.327	vs. 7. year P<0.001	***
		15	6	5.233	0.208	vs. 12. year P=0.965	NS

Figure 5B

dog	*Cl_mito2*	1	3	0.650	0.366	control	-
		3	3	2.189	0.940	vs. 1. year P=0.008	**
		4	3	3.244	0.426	vs. 3. year P=0.002	**
		7	3	4.507	0.887	vs. 4. year P=0.005	**
		9	3	5.356	0.572	vs. 7. Year P<0.001	***
		10	3	6.265	0.280	vs.9. year P<0.001	***
		11	3	6.500	0.586	vs. 10. year P=0.009	**
		12	3	6.675	1.120	vs. 11. year P=0.003	***
		15	3	4.065	1.202	vs. 12. year P=0.856	NS

Figure 5B

Background	Primer	Adult age	Mean of relative 6mA level	±SD	Independent T-test (with Levene's test), P value	Significance level
dog	*Cl_mito2*	young group (1 and 4 years)	2.028	1.065	control	-
		old group (12 and 15 years)	5.747	1.191	vs. control P=0.0025	**

Extended Data Table S2. Statistical data for Figure 3.

Background	Adult age (days)	Trials	6mA level (fmol/1000ng)	±SD	Statistic	Significance level
**Figure 3**						
C. elegans	1	1	0.55	-	-	-
	7	1	3.81	-	-	-
